# Adherence to Follow-up Testing Recommendations in US Veterans Screened for Lung Cancer, 2015-2019

**DOI:** 10.1001/jamanetworkopen.2021.16233

**Published:** 2021-07-08

**Authors:** Eduardo R. Núñez, Tanner J. Caverly, Sanqian Zhang, Mark E. Glickman, Shirley X. Qian, Jacqueline H. Boudreau, Christopher G. Slatore, Donald R. Miller, Renda Soylemez Wiener

**Affiliations:** 1 Center for Healthcare Organization & Implementation Research, Bedford VA Healthcare System, Bedford, Massachusetts; 2 The Pulmonary Center, Boston University School of Medicine, Boston, Massachusetts; 3 VA Boston Healthcare System, Boston, Massachusetts; 4 VA Ann Arbor Healthcare System, Ann Arbor, Michigan; 5 University of Michigan School of Medicine, Ann Arbor; 6 Department of Statistics, Harvard University, Cambridge, Massachusetts; 7 Center to Improve Veteran Involvement in Care, VA Portland Health Care System, Portland, Oregon; 8 Division of Pulmonary and Critical Care Medicine, Oregon Health & Science University, Portland

## Abstract

**Importance:**

Lung cancer screening (LCS) can reduce lung cancer mortality with close follow-up and adherence to management recommendations. Little is known about factors associated with adherence to LCS in real-world practice, with data limited to case series from selected LCS programs.

**Objective:**

To analyze adherence to follow-up based on standardized follow-up recommendations in a national cohort and to identify factors associated with delayed or absent follow-up.

**Design, Setting, and Participants:**

This retrospective cohort study was conducted in Veterans Health Administration (VHA) facilities across the US. Veterans were screened for lung cancer between 2015 to 2019 with sufficient follow-up time to receive recommended evaluation. Patient- and facility-level logistic regression analyses were performed. Data were analyzed from November 26, 2019, to December 16, 2020.

**Main Outcomes and Measures:**

Receipt of the recommended next step after initial LCS according to Lung CT Screening Reporting & Data System (Lung-RADS) category, as captured in VHA or Medicare claims.

**Results:**

Of 28 294 veterans (26 835 [94.8%] men; 21 969 individuals [77.6%] were White; mean [SD] age, 65.2 [5.5] years) who had an initial LCS examination, 17 863 veterans (63.1%) underwent recommended follow-up within the expected timeframe, whereas 3696 veterans (13.1%) underwent late evaluation, and 4439 veterans (15.7%) had no apparent evaluation. Facility-level differences were associated with 9.2% of the observed variation in rates of late or absent evaluation. In multivariable-adjusted models, Black veterans (odds ratio [OR], 1.19 [95% CI, 1.10-1.29]), veterans with posttraumatic stress disorder (OR, 1.13 [95% CI, 1.03-1.23]), veterans with substance use disorders (OR, 1.11 [95% CI, 1.01-1.22]), veterans with lower income (OR, 0.88 [95% CI, 0.79-0.98]), and those living at a greater distance from a VHA facility (OR, 1.06 [95% CI, 1.02-1.10]) were more likely to experience delayed or no follow-up; veterans with higher risk findings (Lung-RADS category 4 vs Lung-RADS category 1: OR, 0.35 [95% CI, 0.28-0.43]) and those screened in high LCS volume facilities (OR, 0.38 [95% CI, 0.21-0.67]) or academic facilities (OR, 0.86 [95% CI, 0.80-0.92]) were less likely to experience delayed or no follow-up. In sensitivity analyses, varying how stringently adherence was defined, expected evaluation ranged from 14 486 veterans (49.7%) under stringent definitions to 20 578 veterans (78.8%) under liberal definitions.

**Conclusions and Relevance:**

In this cohort study that captured follow-up care from the integrated VHA health care system and Medicare, less than two-thirds of patients received timely recommended follow-up after initial LCS, with higher risk of delayed or absent follow-up among marginalized populations, such as Black individuals, individuals with mental health disorders, and individuals with low income, that have long experienced disparities in lung cancer outcomes. Future work should focus on identifying facilities that promote high adherence and disseminating successful strategies to promote equity in LCS among marginalized populations.

## Key Points

**Question:**

What factors are associated with adherence to recommended testing after initial lung cancer screening in the Veterans Health Administration?

**Findings:**

In this retrospective cohort study of 28 294 veterans, more than one-quarter of veterans received delayed or no follow-up testing. Veterans with higher risk findings and those in high-volume or academic centers were more likely to receive timely follow-up, while Black veterans, veterans with mental health disorders, and veterans with lower income were more likely to have delayed or absent follow-up.

**Meaning:**

These findings suggest that even in an integrated health care system, inequities in adherence to lung cancer screening persisted.

## Teaser

This cohort study examines adherence to follow-up testing recommendations after lung cancer screening among US veterans.

## Introduction

Lung cancer remains the leading cause of cancer mortality, with 1.8 million deaths annually worldwide. In the National Lung Screening Trial (NLST), yearly lung cancer screening (LCS) with low-dose computed tomography (CT) achieved a 20% relative reduction in lung cancer mortality.^1^ The NLST benefitted from the healthy volunteer bias, which may be associated with high rates of adherence. Indeed, 95% of NLST participants were adherent with annual screening, and 93% were adherent with recommended evaluation of screen-detected findings. Inability to achieve such high adherence in nontrial settings is likely to reduce the effectiveness of LCS.^2^

Although the US Preventive Services Task Force has recommended LCS for high-risk individuals since 2013,^3^ there has been limited evidence on follow-up after initial LCS in real-world settings, with selected institutions reporting adherence ranging from 37% to 86%.^4,5,6,7,8,9^ Barriers to adherence exist at the patient level (eg, reduced access to preventive health care among smokers),^10,11,12^ clinician level (eg, insufficient knowledge of LCS and pulmonary nodule evaluation),^13,14,15^ and system level (eg, insufficient resources to track and ensure appropriate evaluation).^7,16^

Veterans are at increased risk of lung cancer, given high rates of tobacco use, with a large population of veterans eligible for LCS.^17,18^ Although the Veterans Health Administration (VHA), the largest national integrated health care system in the US, was an early adopter of LCS, subsequent implementation has been inconsistent across VHA facilities.^19^ Thus, the VHA provides an ideal real-world setting to study adherence after initial LCS across the US. We sought to determine adherence to recommended next steps (ie, annual screening or evaluation of screen-detected findings) in a national cohort of veterans screened for lung cancer and identify factors associated with delayed or absent follow-up.

## Methods

This cohort study was approved by the Bedford VA Healthcare System institutional review board. This was an observational study without any direct patient contact and considered to be of minimal risk; therefore, a waiver of informed consent was obtained from the Bedford VA Healthcare System institutional review board. This study followed the Strengthening the Reporting of Observational Studies in Epidemiology (STROBE) reporting guideline for cohort studies.

### Study Design and Population

We conducted a retrospective cohort analysis of veterans ages 55 to 80 years with more than 30 pack-years smoking history and either currently smoking status or quit less than 15 years ago who underwent initial LCS in any VHA facility from January 1, 2015, to November 30, 2019. Using the VHA’s Corporate Data Warehouse (CDW), we identified veterans who had either a health factor for *agrees to screening* tag in the electronic health record (EHR) generated by VHA’s LCS clinical reminder followed by a chest CT within 3 months or a chest CT used for LCS based on *Current Procedural Terminology* codes or CDW radiology tables (eTable 1 in the Supplement). We then narrowed the sample to those whose first LCS (index) CT report included a category (derived from a structured field) based on the American College of Radiology’s Lung CT Screening Reporting & Data System (Lung-RADS), which standardizes reporting of LCS results by categorizing findings according to risk and includes a recommendation for follow-up testing (Table 1).^20^ If the index LCS examination was categorized as Lung-RADS category 0 (ie, incomplete examination; recommend repeat low-dose CT), we used the subsequent LCS examination as the index LCS. Veterans were excluded if they had insufficient follow-up time after index LCS to receive expected evaluation according to Lung-RADS category (eFigure 1 in the Supplement).

**Table 1.** Recommended Next Step on Management Based on Lung-RADS Category and the Expected Time Frame of Evaluation in Our Study Population

Lung-RADS category	Descriptor	Recommended management	Expected time frame of follow-up^a^		
			Primary analysis	Stringent model	Liberal model
0	Incomplete examination	Repeat LDCT	NA^b^		
1	Negative: no nodules and definitely benign nodules	Repeat annual screening LDCT in 1 y	Any CT chest scan 10-15 mo after index LCS	Any CT chest scan 10-13 mo after index LCS	Any CT chest scan 10-24 mo after index LCS
2	Benign: nodules with a very low 90% likelihood of becoming a clinically active cancer owing to size or lack of growth				
3	Probably benign finding(s): short-term follow-up suggested; includes nodules with a low likelihood of becoming a clinically active cancer	Interval chest CT in 6 mo	Any CT chest scan 4-9 mo after index LCS	Any CT chest scan 4-7 mo after index LCS	Any CT chest scan 4-12 mo after index LCS
4A	Suspicious: findings for which additional diagnostic testing and/or tissue sampling is recommended	Interval chest CT or PET in 3 mo	Any CT chest or PET scan 1-5 mo after index LCS	Any CT chest or PET scan 1-4 mo after index LCS	Any CT chest or PET scan 1-6 mo after index LCS
4B or 4X		Interval chest CT or PET or tissue sampling	Any CT chest or PET scan or invasive lung procedure 0-5 mo after index LCS	Any CT chest or PET scan or invasive lung procedure 0-4 mo after index LCS	Any CT chest or PET scan or invasive lung procedure 0-6 mo after index LCS

Abbreviations: CT, computed tomography; LCS, lung cancer screening; LDCT, low-dose CT; Lung-RADS, Lung CT Screening Reporting & Data System; PET, positron emission tomography.

^a^ Evaluation considered early or late if performed before or after expected time frame, respectively.

^b^ For purposes of this study, the repeated LDCT after a Lung-RADS 0 result was treated as index LCS.

### Primary Outcome

The primary outcome was receipt of the recommended next step after index LCS according to Lung-RADS category. We queried VHA CDW through February 29, 2020 and Medicare claims data (available through December 31, 2018) for evidence of expected follow-up tests, including chest CT, positron emission tomography, or invasive procedures, defined by *International Classification of Diseases, Ninth Revision* (*ICD-9*)^21^ and *Tenth Revision* (ICD-10)^22^ and *Current Procedural Terminology* codes (eTable 1 and eTable 2 in the Supplement). The timeliness of evaluation was defined by Lung-RADS category. Similar to NLST, for our primary analysis, we defined expected evaluation as occurring within 2 months before or 3 months after the recommended timeframe, except for Lung-RADS category 4, which used 2 months after as the cutoff (Table 1). Veterans were considered to have early or late follow-up if the recommended test occurred before or after the expected timeframe, respectively. Veterans were categorized as receiving no evaluation if they had sufficient follow-up time after index LCS to reach the end of the expected evaluation period, but no follow-up testing was found.

### Covariables

We a priori selected clinically relevant covariables based on prior studies of adherence to cancer screenings, including self-reported race/ethnicity.^23,24,25,26^ For each veteran, we extracted data from VHA CDW on demographic characteristics and VHA priority status (ie, VHA’s system to determine copays). We used the veteran’s residential zip code to estimate distance to nearest VHA facility, median income, and urbanization using the US Census Bureau’s rural-urban commuting area codes.^27^ We defined comorbidities based on *ICD-9* and *ICD-10* codes in VHA CDW or Medicare files and calculated Elixhauser comorbidity index scores.^28,29^ As a measure of health care utilization, the number of VHA outpatient visits in the year prior to index LCS was collected for each veteran. We performed log transformation of covariates that did not follow a normal distribution, including outpatient visits, median income, and distance to VHA facility.

We assigned veterans to VHA facilities based on where they received the most outpatient visits in the year prior to index LCS. Facility-level covariates included academic status based on graduate medical education affiliations, US census region, and LCS volume.^30,31^ We used data from a national VHA survey conducted in 2013 to 2014 to determine whether facilities had capacity to perform thoracic surgery.^32^

### Statistical Analysis

We conducted mixed-effects logistic regression analyses, including patient- and site-level variables, to determine factors associated with delayed or absent follow-up after index LCS compared with early or expected follow-up. Our models assumed that coefficients to patient-level variables were random effects that varied by site. We report the mean patient outcomes, accounting for site-specific random effects, in our 2-sided analytic testing. We a priori selected *P* = .05 as statistically significant. To address potential issues of multicollinearity, we computed variance inflation factors for each patient-level variable in the model. No variables had variance inflation factors greater than 10, a conventional threshold for collinearity concerns, suggesting that collinearity was not problematic.^33^ To quantify facility-level variation, we calculated an intraclass correlation coefficient on the model with patient fixed effects and random intercepts that varied by site.^34^ All analyses were performed using SAS statistical software version 9.4 (SAS Institute). Data were analyzed from November 26, 2019, to December 16, 2020.

#### Sensitivity Analyses

We performed sensitivity analyses to examine different definitions of adherence and to check the validity of our conclusions about factors associated with adherence to Lung-RADS recommendations. First, to explore the potential for error introduced into the model by creating cutoffs for adherence, we created 2 models with varying periods for expected evaluation, one more stringent than that used by NLST and one more liberal. All models defined early evaluation as follow-up testing occurring greater than 2 months before the recommended timeframe, to maintain consistency (Table 1). In the stringent adherence model, we defined expected evaluation as occurring within 2 months before or 1 month after the recommended timeframe for all Lung-RADS categories (eg, for Lung-RADS categories 1 and 2, follow-up is recommended at 12 months, so follow-up within 10-13 months would fall within the expected evaluation). In the liberal model, we defined expected evaluation as occurring within 24 months of index LCS for Lung-RADS categories 1 and 2, 12 months for Lung-RADS category 3, and 6 months for Lung-RADS category 4, following expert consensus of acceptable evaluation delays during the COVID-19 pandemic.^35^

To explore the outcomes associated with combining low-risk findings (Lung-RADS categories 1 and 2) with higher risk findings (Lung-RADS categories 3 and 4) into our model, which may have different mechanisms of adherence, we conducted a sensitivity analysis stratifying our regression model by low and high-risk findings. To account for potential error introduced by lack of evaluation among veterans with limited life expectancy, who are unlikely to benefit from LCS and may have elected to decline evaluation, we performed a sensitivity analysis excluding veterans with Care Assessment Needs score of 95 or greater. The validated Care Assessment Needs score is calculated in the VHA EHR to identify veterans with increased risk of hospitalization and death, and a score of 95 or greater is 89% specific for frailty and correlates with an approximately 20% risk of death within 1 year.^36^ Because we were unable to capture care provided through private insurers, we performed a sensitivity analysis restricting our analysis to Medicare-eligible veterans (ie, age ≥65 years), as veterans with VHA or Medicare coverage may be less likely to obtain screening through the private sector.^37,38^ Additionally, because Medicare claims data are only available through 2018, we performed a sensitivity analysis restricting our study period to January 1, 2015, to December, 31, 2018.

#### EHR Review

To further explore the ability of our claims-based algorithm to detect recommended evaluation, a pulmonologist (E.R.N.) performed detailed EHR reviews on a randomly selected sample of veterans with low-risk (Lung-RADS category 1 or 2; 40 veterans) and intermediate risk (Lung-RADS category 3 or 4A; 40 veterans) findings who received no apparent evaluation; as well as all veterans with Lung RADS category 4B or 4X findings (42 veterans) with no subsequent follow-up captured through VHA or Medicare claims.

## Results

Overall, 35 699 veterans had initial LCS examinations with Lung-RADS recommendations; 7405 veterans (20.7%) were excluded owing to insufficient time to determine adherence to follow-up testing (eTable 3 in the Supplement). Our final sample included 28 294 veterans (26 835 [94.8%] men; 21 969 individuals [77.6%] were White; mean [SD] age, 65.2 [5.5] years) (Table 2). There were moderate rates of chronic obstructive pulmonary disease (COPD) (9667 veterans [34.2%]) and mental health conditions (eg, depression: 7370 veterans [26.0%]). Most veterans (21 557 veterans [76.2%]) had low-risk findings on the index LCS (ie, Lung-RADS category 1 or 2), while 4001 veterans (14.1%) had indeterminate results (ie, Lung-RADS category 3), and 2736 veterans (9.7%) had suspicious findings (ie, Lung-RADS category 4).

**Table 2.** Characteristics of Veterans and VHA Facilities

Characteristic	No. (%) (N = 28 294)^a^
**Individual level**	
Age, mean (SD), y	65.2 (5.5)
Sex	
Men	26 835 (94.8)
Women	1459 (5.2)
Race/ethnicity	
White	21 969 (77.6)
Black	5210 (18.4)
Hispanic	602 (2.1)
Other^b^	513 (1.8)
Married	12 225 (43.2)
Zip code–level income, median (IQR), $	46 306 (36 910-55 702)
Distance from home to LCS facility, median (IQR), mi	28.2 (2.6-53.8)
Live in rural zip code	6053 (21.4)
VHA benefits (priority status)	
Highly disabled	8143 (28.8)
Low or moderately disabled	6560 (23.2)
Limited with copayments	3698 (13.1)
Poverty with no copayments	9892 (35.0)
Comorbid conditions	
Chronic obstructive lung disease	9667 (34.2)
Congestive heart failure	1731 (6.1)
History of major adverse cardiac event	3119 (11.0)
Chronic kidney disease	2918 (10.3)
Dementia	731 (2.6)
Depression	7370 (26.0)
Anxiety	3737 (13.2)
PTSD	4701 (16.6)
Schizophrenia	706 (2.5)
Substance use disorder	7590 (26.8)
Elixhauser comorbidity index, mean (SD)	4.2 (3.1)
Outpatient visits 1 y before LCS, median (IQR), No.	14 (5.5-22.5)
**Facility level**^**c**^	
US census region	
Northeast	2252 (8.0)
Midwest	6793 (24.0)
South	15 633 (55.2)
West	3611 (12.8)
Academic	17 805 (62.9)
Thoracic surgery available	26 221 (92.7)
LCSs performed, No.	
<500	6738 (23.8)
500-1000	10 665 (37.7)
>1000	10 891 (38.5)

Abbreviations: IQR, interquartile range; LCS, lung cancer screening; PTSD, posttraumatic stress disorder; VHA, Veterans Health Administration.

^a^ Variables with missing data include race (461 veterans [1.6%]), marital status (164 veterans [0.6%]), distance to nearest VHA facility (589 veterans [2.1%]), median income (519 veterans [1.8%]), thoracic surgery availability at preferred VHA facility (1136 veterans [4.0%]).

^b^ Other race/ethnicity included primarily Asian and American Indian or Alaska Native individuals.

^c^ Based on preferred VHA facility where veteran received the most outpatient visits in the year prior to index LCS.

**Figure 1.**
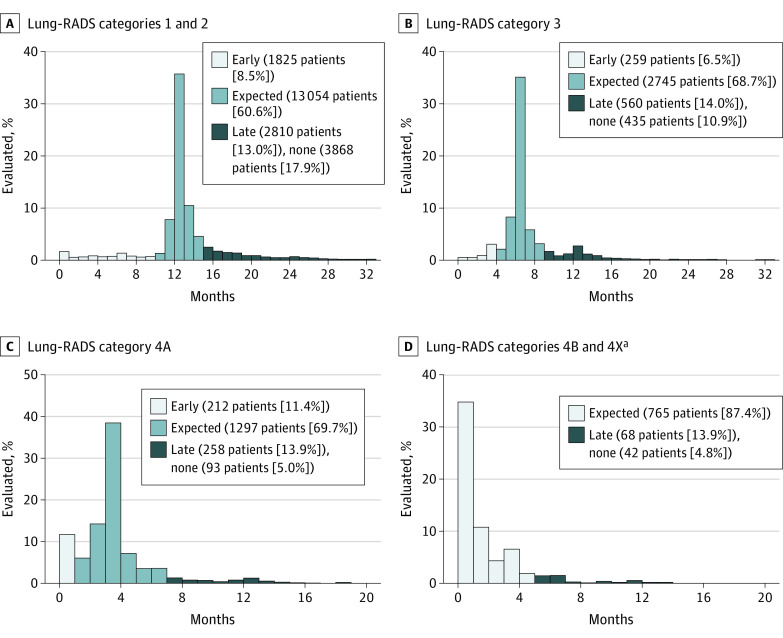
Adherence to Lung-RADS Recommendation Stratified by Lung-RADS Category Expected adherence was defined as receiving follow-up evaluation within 2 months before or 1 month after the recommended time frame; early adherence was considered an evaluation before that period, and late adherence was considered an evaluation after that period. ^a^Early adherence is not applicable to Lung-RADS 4A and 4X, as recommended evaluation timeframe is 0 to 3 months.

A total of 23 855 veterans (84.3%) had evidence of subsequent testing after the index LCS, with 2296 veterans (8.1%) receiving early testing, 17 863 veterans (63.1%) receiving testing in the expected timeframe, and 3696 veterans (13.1%) receiving delayed testing (Table 3). Of note, 4439 veterans (15.7%) received no apparent follow-up. When stratified by Lung-RADS category, veterans with higher-risk findings had lower rates of no evaluation (3868 veterans [17.9%] with Lung-RADS category 1 or 2; 435 veterans [10.9%] with Lung-RADS category 3; 250 veterans [13.9%] with Lung-RADS category 4A; 42 veterans [4.8%] with Lung-RADS category 4B or 4X), whereas delayed follow-up occurred in 10% to 20% of veterans across Lung-RADS categories (Figure 1).

**Table 3.** Model Results of Primary Analysis of Adherence vs Models Using Alternate Definitions of Expected Follow-up and Sensitivity Analyses

Analysis	Evaluation, No. (%)				Total, No.
	Early	Expected	Late	No evaluation	
Primary^a^	2296 (8.1)	17 863 (63.1)	3696 (13.1)	4439 (15.7)	28 294
Alternate definitions of adherence^b^					
Stringent	2418 (8.3)	14 486 (49.7)	6951 (23.9)	5301 (18.2)	29 137
Liberal	1986 (7.6)	20 579 (78.8)	1290 (4.9)	2259 (8.7)	26 114
Sensitivity					
Exclude veterans with CAN score >95^c^	2254 (8.0)	17 737 (63.2)	3662 (13.0)	4415 (15.7)	28 068
Exclude veterans age <65 y	1403 (8.9)	10 297 (65.4)	1863 (11.8)	2173 (13.8)	15 736
Restricted to study period 2015-2018	1273 (8.8)	9306 (64.5)	1500 (10.4)	2350 (16.3)	14 429

Abbreviations: CAN, Care Assessment Needs; Lung-RADS, Lung CT Screening Reporting & Data System.

^a^ In our primary analysis, expected follow-up is defined as occurring within 3 months of the recommended Lung-RADS evaluation for Lung-RADS 1-3 and within 2 months for Lung-RADS 4. All models defined an early evaluation as occurring more than 2 months before the recommended Lung-RADS.

^b^ In the stringent model, expected follow-up is defined as occurring within 1 month of the Lung-RADS recommended interval; in the liberal model, expected follow-up is defined as occurring within 12 months of the recommended evaluation for Lung-RADS 1 and 2, 6 months for Lung-RADS 3, and 3 months for Lung-RADS 4.

^c^ CAN score of 95 or greater indicates estimated 20% risk of death within 1 year.

**Figure 2.**
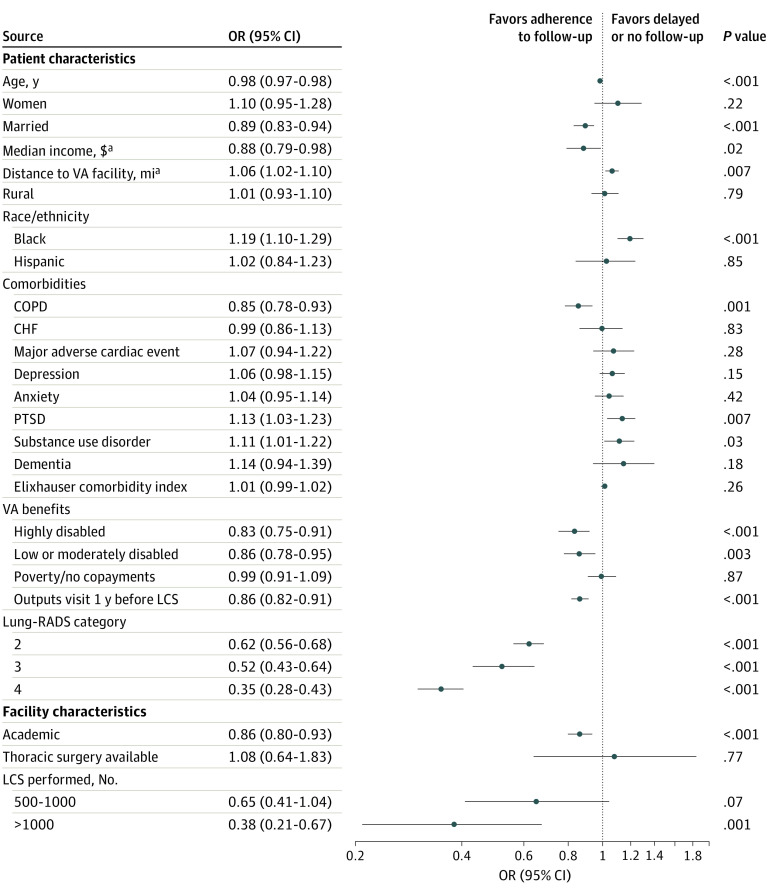
Patient- and Facility-Level Factors Associated With Delayed or No Adherence to Lung CT Screening Reporting & Data System (Lung-RADS) Recommendations Covariables included in the model that are not reported here include: race (other), comorbidities (chronic kidney disease, schizophrenia, HIV), and facility characteristics geographic location (East, South, Midwest, West). Full model outputs are provided in eTable 3 in the Supplement. CHF indicates congestive heart failure; COPD, chronic obstructive pulmonary disease; LCS, lung cancer screening; PTSD, posttraumatic stress disorder. ^a^Reflects log-transformed variable.

In our primary multivariable analysis, veterans with high-risk findings were less likely to have delayed or no evaluation (Lung-RADS category 4 vs Lung-RADS category 1: odds ratio [OR], 0.35 [95% CI, 0.28-0.43]) (Figure 2). Black veterans, compared with White veterans, were more likely to have delayed or no evaluation (OR, 1.19 [95% CI, 1.10-1.29]); by contrast, veterans with higher income (OR, 0.88 [95% CI, 0.79-0.98]) and married veterans (OR, 0.89 [95% CI, 0.83-0.94]) were less likely to experience delayed or absent evaluation. While veterans with COPD were less likely to experience delayed or absent testing (OR, 0.85 [95% CI, 0.78-0.93]), veterans were more likely to have delayed or absent evaluation if they had mental health comorbidities, including posttraumatic stress disorder (OR, 1.13 [95% CI, 1.03-1.23]) or substance use disorder (OR, 1.11 [95% CI, 1.01-1.22]).

Veterans classified as severely disabled who had no copays (OR, 0.83 [95% CI, 0.75-0.91]) and those with partial disability with reduced copays (OR, 0.86 [95% CI, 0.78-0.95]) were less likely to have delayed or absent follow-up compared with veterans with copays to access VHA care. Additionally, veterans who had fewer outpatient visits or lived farther from a VHA facility were more likely to experience delayed or absent evaluation (Figure 2). The log-transformed covariates had similar interpretations: for every doubling in number of outpatient visits (OR, 0.86 [95% CI, 0.82-0.91]) or miles to VHA facility (OR, 1.06 [95% CI, 1.02-1.10), there was a decrease in the odds of having delayed or absent follow-up.

A total of 122 facilities performed at least 1 index LCS examination in the study period. Veterans screened in high-LCS volume (ie, >1000 LCSs during study period) facilities were less likely to have delayed or missing follow-up (OR, 0.38 [95% CI, 0.21-0.67]), as were those screened in academic facilities (OR, 0.86 [95% CI, 0.80-0.92]) (Figure 2). Our multivariable model with intercepts varying by site had an intraclass correlation coefficient of 0.092, suggesting that 9.2% of the variation in adherence to LCS was attributable to facilities rather than differences in the veterans who received care at those facilities. Additionally, to explore variation of patient-level outcomes by facility, we examined the variation across facilities in odds of delayed or no follow-up by Lung-RADS categories (eFigure 2 in the Supplement).

Using alternate definitions of adherence resulted in expected follow-up ranging from 14 486 veterans (49.7%) in the stringent model to 20 579 veterans (78.8%) in the liberal model, indicating that rates of adherence can vary substantially depending on how adherence is defined (Table 3). Other sensitivity analyses showed only a small change in proportion of veterans without evaluation when excluding those ineligible for Medicare (4415 veterans [15.7%] vs 2173 veterans [13.8%]) but no difference when excluding veterans with high projected mortality or when restricting the study period to fiscal years 2015 to 2018 (Table 3). There were no changes in the direction of covariate estimates in multivariable models using alternative definitions of adherence (stringent or liberal) or in models separating low-risk (ie, Lung-RADS categories 1 and 2) and high-risk (ie, Lung-RADS categories 3 and 4) findings (eTable 4 in the Supplement).

EHR review of randomly selected veterans with low-risk or intermediate-risk findings with no apparent follow-up (based on claims data) confirmed absence of follow-up in 38 of 40 veterans (95.0%) with Lung-RADS category 1 or 2 findings and 36 of 40 veterans (90.0%) with Lung-RADS category 3 or 4 findings. By contrast, of 42 veterans with Lung-RADS category 4B or 4X findings with no apparent follow-up, 16 veterans (38.1%) were found on EHR review to have received evaluation outside of the VHA that our study did not capture (eg, care obtained through private insurer). Of the remaining 26 veterans with 4B or 4X findings, the most common reasons for lack of follow up included patient declined follow-up (7 veterans [16.7%]), several attempts but unable to schedule (10 veterans [23.8%]), and no documented follow-up (8 veterans [19.0%]).

## Discussion

In this national cohort study of 28 294 veterans who underwent initial LCS, we found suboptimal adherence to Lung-RADS recommendations, with approximately 13% receiving late evaluation and approximately 16% receiving no apparent follow-up. Our study, which captured follow-up testing from VHA and Medicare systems, illustrates the challenges of providing timely, recommended next steps after LCS in real-world practice. The mortality benefit of LCS observed in the NLST^1^ and NELSON trials^39^ could in part be attributed to high adherence (>90%) to timely, appropriate follow-up after initial screening. In real-world practice, in addition to documented low uptake of initial LCS (estimated at 4%-14% of the eligible US population^40,41,42^), suboptimal adherence to follow-up evaluations could further erode the potential of LCS to reduce lung cancer death.

At the patient level, veterans with higher-risk Lung-RADS categories were significantly more like to receive adherent follow-up care. This is reassuring, given that timely evaluation of high-risk findings is essential to identifying cancers at an early, treatable stage, thereby avoiding disease progression and improving lung cancer outcomes.^43,44,45,46^ But we identified substantial variation across facilities, suggesting there is room for further improvement in some facilities. Indeed, veterans screened in academically affiliated or high LCS–volume facilities were less likely to have delayed or no follow-up, suggesting lessons could be learned from these LCS programs. Based on the included variables, our model identified a moderate amount of facility-level variation.^47^ It is likely that additional facility-level LCS infrastructure that we could not capture may be associated with improved adherence. For example, single institution studies suggest LCS coordinators and tracking systems, which are components recommended by the American Thoracic Society and American College of Chest Physicians, may improve adherence and timeliness of evaluation.^48^ Yet, even when facilities implement LCS coordinators and tracking software, significant interfacility variation in adherence remains.^9^ Thus, future work should evaluate LCS programs with high vs low rates of adherence to identify and spread best practices for timely, appropriate follow-up.

Disparities in cancer screening and lung cancer outcomes are typically decreased in VHA and military settings compared with the private sector,^49,50,51^ which may be owing to the more uniform access to care typically experienced in integrated health systems. Yet, we found disparities in receiving the recommended next steps after LCS among marginalized populations, such as Black veterans and those with lower income, who were more likely to experience delayed or absent evaluation, reflecting the well-established finding of disparities in cancer care among those from underrepresented ethnic and racial backgrounds or lower socioeconomic status.^52,53,54^ We found that veterans with posttraumatic stress disorder and substance use disorders were more likely to have delayed or no follow-up, likely reflecting mental health barriers identified in other cancer screenings.^55,56^ Veterans with fewer VHA outpatient visits in the prior year, higher copays, and longer distances to travel to access VHA care were more likely to have delayed or absent follow-up after initial LCS. Prior studies illustrate the importance of health care utilization, insurance, and access to care as critical to ensuring appropriate cancer screening and reducing cancer mortality.^57,58,59,60,61^ Unfortunately, LCS access is not evenly distributed across the VHA system.^62^ Therefore, it is imperative for the VHA to continue to allocate resources to improve LCS access and adherence to reduce lung cancer mortality in high-risk veteran populations, with particular attention to marginalized groups, such as Black veterans, those with mental health conditions, and those with socioeconomic barriers to receiving appropriate and timely care.

### Limitations

This study has some limitations. First, we relied on administrative data, which can result in misclassified or missed data if there are errors in coding of LCS examinations or follow-up tests. We were unable to capture care received outside of VHA or Medicare, which may have resulted in an underestimate of adherence if substantial care was received through private insurers. However, our sensitivity analysis excluding veterans younger than 65 years who are more likely to be dual users of VHA and private insurance showed only minor differences in follow-up evaluation. Moreover, our EHR review of randomly selected veterans with Lung-RADS categories 1 through 4A findings, who constituted 97% of our cohort, showed our algorithm missed follow-up testing in less than 10% of veterans. Of note, our results may not be generalizable outside the VHA or among nonveteran populations. In particular, the VHA’s national integrated health care system has well-established processes to facilitate guideline-recommended, multidisciplinary cancer care and pulmonary nodule evaluation, and achieves equivalent or better quality of cancer care and survival than the private sector.^63,64,65,66,67,68^ Thus the 63% rate of expected evaluation identified in this study may exceed that anticipated in other settings. Additionally, we were unable to measure patient-centered outcomes, such as lung cancer diagnoses and mortality, and instead focus on adherence to screening, which has been shown to be a key factor in lung cancer mortality.^2^

## Conclusions

To our knowledge, this cohort study represents the first national analysis of adherence to Lung-RADS recommendations among a large cohort with diverse geographic distribution and socioeconomic status. We found that even in an integrated health care system, many patients did not receive timely recommended follow-up after initial LCS. While it is encouraging that veterans with the highest risk findings were more likely to receive recommended follow-up care, disparities in timely follow-up were demonstrated among marginalized populations who have long experienced worse lung cancer outcomes, such as Black veterans and those with low income. To optimize the mortality benefit of LCS, it is clear that further work must be done to improve adherence overall and especially among marginalized populations. Future work should focus on learning effective strategies from LCS centers with high adherence to extend successful program features to the people and locations with greater barriers to care.

## Notes

Supplement. **eTable 1.** *ICD-9*, *ICD-10*, and *CPT* Codes for Lung Cancer Screening Analysis of Procedures **eTable 2.** *ICD-9*, *ICD-10*, and *CPT* Codes for Lung Cancer Screening Analysis of Comorbidities **eFigure 1.** Cohort Derivation by Primary Analysis Adherence and Stringent and Liberal Adherence Definitions **eTable 3.** Baseline Characteristics of Veterans Included in Study and Excluded Owing to Insufficient Time to Determine Adherence to Follow-up Testing **eFigure 2.** Individual Facility Odds of Delayed or No Evaluation Stratified by Lung-RADS Group Compared With Lung-RADS 1 **eTable 4.** Multivariable Logistic Regression Analyses of the Study Cohort Compared With Using Strict and Liberal Adherence Time Cutoffs and Comparing Primary Study Cohort With Low-Risk and High-Risk Separated
