# Supplementary material for: Adherence to Follow-up Testing Recommendations in US Veterans Screened for Lung Cancer, 2015-2019
Source: JAMA Netw Open. 2021 Jul 8;4(7):e2116233. doi: 10.1001/jamanetworkopen.2021.16233 (PMC8267608; doi:10.1001/jamanetworkopen.2021.16233)
Supplement: Supplement. — eTable 1. ICD-9, ICD-10, and CPT Codes for Lung Cancer Screening Analysis of Procedures eTable 2. ICD-9, ICD-10, and CPT Codes for Lung Cancer Screening Analysis of Comorbidities eFigure 1. Cohort Derivation by Primary Analysis Adherence and Stringent and Liberal Adherence Definitions eTable 3. Baseline Characteristics of Veterans Included in Study and Excluded Owing to Insufficient Time to Determine Adherence to Follow-up Testing eFigure 2. Individual Facility Odds of Delayed or No Evaluation Stratified by Lung-RADS Group Compared With Lung-RADS 1 eTable 4. Multivariable Logistic Regression Analyses of the Study Cohort Compared With Using Strict and Liberal Adherence Time Cutoffs and Comparing Primary Study Cohort With Low-Risk and High-Risk Separated [file jamanetwopen-e2116233-s001.pdf]

# Supplemental Online Content

Núñez ER, Caverly TJ, Zhang S, et al. Adherence to follow-up testing recommendations in US veterans screened for lung cancer, 2015-2019. *JAMA Netw Open*. 2021;4(7):e2116233.
doi:10.1001/jamanetworkopen.2021.16233

**eTable 1.** *ICD-9*, *ICD-10*, and *CPT* Codes for Lung Cancer Screening Analysis of Procedures

**eTable 2.** *ICD-9*, *ICD-10*, and *CPT* Codes for Lung Cancer Screening Analysis of Comorbidities

**eFigure 1.** Cohort Derivation by Primary Analysis Adherence and Stringent and Liberal Adherence Definitions

**eTable 3.** Baseline Characteristics of Veterans Included in Study and Excluded Owing to Insufficient Time to Determine Adherence to Follow-up Testing

**eFigure 2.** Individual Facility Odds of Delayed or No Evaluation Stratified by Lung-RADS Group Compared With Lung-RADS 1

**eTable 4.** Multivariable Logistic Regression Analyses of the Study Cohort Compared With Using Strict and Liberal Adherence Time Cutoffs and Comparing Primary Study Cohort With Low-Risk and High-Risk Separated

This supplemental material has been provided by the authors to give readers additional information about their work.

**eTable 1**. *ICD-9*, *ICD-10*, and *CPT* Codes for Lung Cancer Screening Analysis of Procedures

| Procedure | ICD-9 procedure codes | ICD-10 procedure codes | CPT procedure codes |
|---|---|---|---|
| Transthoracic Needle Lung Biopsy | 33.26 | 0W9C3ZX, 0WBC3ZX, 0B9C3ZX 0B9D3ZX 0B9F3ZX 0B9G3ZX 0B9H3ZX 0B9J3ZX 0B9K3ZX 0B9L3ZX 0B9M3ZX 0BBC3ZX 0BBD3ZX 0BBF3ZX 0BBG3ZX 0BBH3ZX 0BBJ3ZX 0BBK3ZX 0BBL3ZX 0BBM3ZX | 32405 |
| Lung surgery for pulmonary nodule including Open or thorascopic: wedge, segment, lobectomy, pneumonectomy | 32.0, 32.01, 32.09, 32.1, 32.2, 32.20, 32.39, 32.3, 32.30, 32.39, 32.4, 32.41, 32.49, 32.5, 32.50, 32.59, 33.2, 33.20, 33.25, 33.28, | 0BBC4ZX 0BBD4ZX 0BBF4ZX 0BBG4ZX 0BBH4ZX 0BBJ4ZX 0BBK4ZX 0BBL4ZX 0BBM4ZX 0BDC4ZX 0BDD4ZX 0BDF4ZX 0BDG4ZX 0BDH4ZX 0BDJ4ZX 0BDK4ZX 0BDL4ZX, 0BBM0ZZ 0BBM3ZZ 0BBM7ZZ 0BTK4ZZ 0BTL4ZZ 0BTM4ZZ 0BBK4ZZ, 0BBL4ZZ, 0B5K0ZZ 0B5K3ZZ 0B5K7ZZ 0B5L0ZZ 0B5L3ZZ 0B5L7ZZ 0B5M0ZZ 0B5M3ZZ 0B5M7ZZ 0BBK0ZZ 0BBK3ZZ 0BBK7ZZ 0BBL0ZZ 0BBL3ZZ 0BBL7ZZ 0BTG0ZZ 0BTJ0ZZ, 0BB77ZZ 0BB80ZZ0BB83ZZ 0BB87ZZ 0BB90ZZ 0BB93ZZ 0BB97ZZ 0BBB0ZZ 0BBB3ZZ 0BBB7ZZ 0BTC4ZZ 0BTD4ZZ 0BTF4ZZ 0BTG4ZZ 0BTJ4ZZ 0BTC0ZZ 0BTD0ZZ 0BTF0ZZ , B5B0ZZ 0B5B3ZZ 0B5B7ZZ 0BB30ZZ 0BB33ZZ 0BB37ZZ 0BB40ZZ 0BB43ZZ 0BB47ZZ 0BB50ZZ 0BB53ZZ 0BB57ZZ 0BB60ZZ 0BB63ZZ 0BB67ZZ 0BB70ZZ 0BB73ZZ, 0B540ZZ 0B543ZZ 0B547ZZ 0B550ZZ 0B553ZZ 0B557ZZ 0B560ZZ 0B563ZZ 0B567ZZ 0B570ZZ 0B573ZZ 0B577ZZ 0B580ZZ 0B583ZZ 0B587ZZ 0B590ZZ 0B593ZZ 0B597ZZ 0BTG0ZZ, 0BTG0ZZ, 0BTJ0ZZ, 0BTK0ZZ, 0BTK0ZZ, 0BTL0ZZ, 0BTM0ZZ, 0B9L0ZX 0B9M0ZX 0BBK0ZX 0BBL0ZX 0BBM0ZX 0BB10ZZ 07JL0ZZ 07JM0ZZ 0WJ90ZZ 0WJB0ZZ 0WJQ0ZZ, 0BTK0ZZ, 0BTL0ZZ, 0BTM0ZZ, 0BTC0ZZ 0BTD0ZZ, 0BTF0ZZ, 0B930ZX 0B940ZX 0B950ZX 0B960ZX 0B970ZX 0B980ZX 0B990ZX 0B9B0ZX 0BB30ZX 0BB40ZX 0BB50ZX 0BB60ZX 0BB70ZX 0BB80ZX 0BB90ZX 0BBB0ZX 0B9K0ZX 0BDM4ZX 0B9C4ZX 0B9D4ZX 0B9F4ZX 0B9G4ZX 0B9H4ZX 0B9J4ZX 0B9K4ZX 0B9L4ZX 0B9M4ZX, 0BT30ZZ 0BT34ZZ 0BT40ZZ 0BT44ZZ 0BT50ZZ 0BT54ZZ 0BT60ZZ 0BT64ZZ 0BT70ZZ 0BT74ZZ 0BT80ZZ 0BT84ZZ 0BT90ZZ 0BT94ZZ 0BTB0ZZ 0B530ZZ 0B533ZZ 0B537ZZ | 31786, 32095, 32100, 32440, 32442, 32445, 32480, 32482, 32484, 32486, 32488, 32500-32501, 32503-32504, 32520, 32602, 32657, 32663 |
| Mediastinoscopy | 34.22, 34.26 | 0W9C3ZX, 0W9C4ZX, 07B70ZZ, 07BL0ZZ, 07T70ZZ, 0W9C0ZX,0WBC0ZX, 0WCC0ZZ, 0WBC3ZZ, 0WJQ0ZZ, 02BN0ZX, 0W9C0ZZ, 07B70ZZ, 07BL0ZZ, 07T70ZZ | 32605-32606, 32662, 38746, 39000, 39010, 39220, 39400 |
| Bronchoscopy | 33.21-33.24, 33.27 | 0BJ08ZZ, 0BJ08ZZ, 0BJ18ZZ, 0BJK8ZZ, 0BJL8ZZ, 3E1F78Z, 3E1F88X, 3E1F88Z, 0BB28ZX, 0BB38ZX, 0BB48ZX, 0BB58ZX, 0BB68ZX, 0BB78ZX, 0BB88ZX, 0BB98ZX, 0BBB8ZX, 0BD28ZX, 0BD38ZX, 0BD48ZX, 0BD58ZX, 0BD68ZX, 0BD78ZX, 0BD88ZX, 0BD98ZX, 0BDB8ZX, 0B918ZX, 0B928ZX, 0B938ZX, 0B948ZX, 0B958ZX, 0B968ZX, 0B978ZX, 0B988ZX, 0B998ZX, 0B9B8ZX, 0BC18ZZ, 0BC28ZZ, 0BC38ZZ, 0BC48ZZ, 0BC58ZZ, 0BC68ZZ, 0BC78ZZ, 0BC88ZZ, 0BC98ZZ, 0BCB8ZZ, 0BCC8ZZ, 0BCD8ZZ, 0BCG4ZZ, 0BCH4ZZ, 0BCJ4ZZ, 0BCN4ZZ | 31615, 31620, 31622-31625, 31628-31633, 31635-31638, 31640-31641, 31643, 31645-31646, 31652-31654 |

Abbreviations: *ICD*, *International Classification of Diseases*; CPT, Current Procedural Terminology.

© 2021 Núñez ER et al. *JAMA Network Open.*

**eTable 2**. *ICD-9*, *ICD-10*, and *CPT* Codes for Lung Cancer Screening Analysis of Comorbidities

| **Comorbidities** | ICD-9 codes | ICD-10 codes | ICD-9 procedures | ICD-10 procedures | CPT procedure codes |
|---|---|---|---|---|---|
| ***The next 2 variables are based on a single principal code from inpatient records (VA or Medicare) or primary code from Emergency room data anytime before or on the index date.*** | | | | | |
| Major Adverse Cardiac Event | 410, 410.xx, 411.0, 411.1, 411.81, 411.89, 412, 412.xx, 427.5 | I20.0, I21, I21.x, I21.xx, I22.x, I24.0, I24.1, I24.8, I24.9, I25.2, I46.9 | 36.01-36.07, 36.09-36.16, 36.19 | 0210.x, 02111.x, 0212.x, 0213.x, 0270.x, 0271.x, 0272.x, 0273.x | 33510-33519, 33521-33523, 33533-33536, 33572, 92973-92975, 92977, 92980-92982, 92984, 92986, 92995, 92996 |
| ***All other morbidity variables are based on 2+ days with codes (IP or OP, VA or Medicare) in the 731 days (2 years) before and including the index date, except as noted.*** | | | | | |
| COPD | 491.xx, 492.xx, 496.xx | J40-J44.xx | | | |
| Congestive heart failure | 428,428.xx,402.01,402.11,402.91,404.01,404.03, 404.11, 404.13, 404.91, 404.93 | I50.x, I50.xx, I11.0, I13.0, I13.2 | | | |
| Chronic kidney disease | 250.4, 581-583.xx, 585-587.xx, 996.73, 996.81, V42.0, V45.1 | E11.29, E10.29, E11.21, E10.21, N03.x, N04.x, N05.x, N07.x, N08.x, N17.x, N18.x, N19.x, N26.x, T82.8xxA, Z94.0, Z99.2, Z91.15 | | | |
| HIV | 042- 044.xx | B20, B17.1x, B17.8, B17.9, B18.2, B18.8, B18.9 | | | |
| Dementia | 290, 290.xx, 331.0-331.82, 331.9, 331.9x, 797 | F01.5, F01.5x, F02.8, F02.8x, F03.9, F03.9x, G30.x, G31.x, G31.xx, R41.81 | | | |
| Depression | 296.2-296.3x, 300.4, 300.4x, 300.9, 300.9x, 301.12, 309.0, 309.0x, 309.1, 309.1x, 309.28, 311, 311.x | F32.x, F32.xx, F33.x, F33.xx, F34.1, F43.21, F43.23, F48.9 | | | |
| Post-traumatic stress disorder | 309.81 | F43.10, F43.11, F43.12 | | | |
| Schizophrenia | 295, 295.xx | F20.x, F20.xx | | | |
| Substance Use Disorder | 291, 291.xx, 292.0, 292.0x, 292.89, 292.9, 292.9x, 303, 303.xx, 304, 304.xx, 305, 305.0, 305.0x, 305.2-305.9x, 357.5, 425.5, 535.3, 571.0-571.3x, 760.71, 790.3, 977.3, 980.0, 980.9, E860-E860.1, E860.9, E947.3, V11.3, V79.1 | F10-F16.xx, F18-F19.xx, F62.1, I42.6, K29.20, K29.21, K70.x, K70.xx, P04.3, Q86.0, R78.0, T50.991A, T51.0X1A, T51.0X2A, T51.0X3A,T51.0X4A, T51.91XA, T51.92XA, T51.93XA, T51.94XA, NOD.X, Z65.8, Z13.89 | | | |

Abbreviations: *ICD*, *International Classification of Diseases*; CPT, Current Procedural Terminology.

© 2021 Núñez ER et al. *JAMA Network Open.*

**eFigure 1.** Cohort Derivation by Primary Analysis Adherence and Stringent and Liberal Adherence Definitions

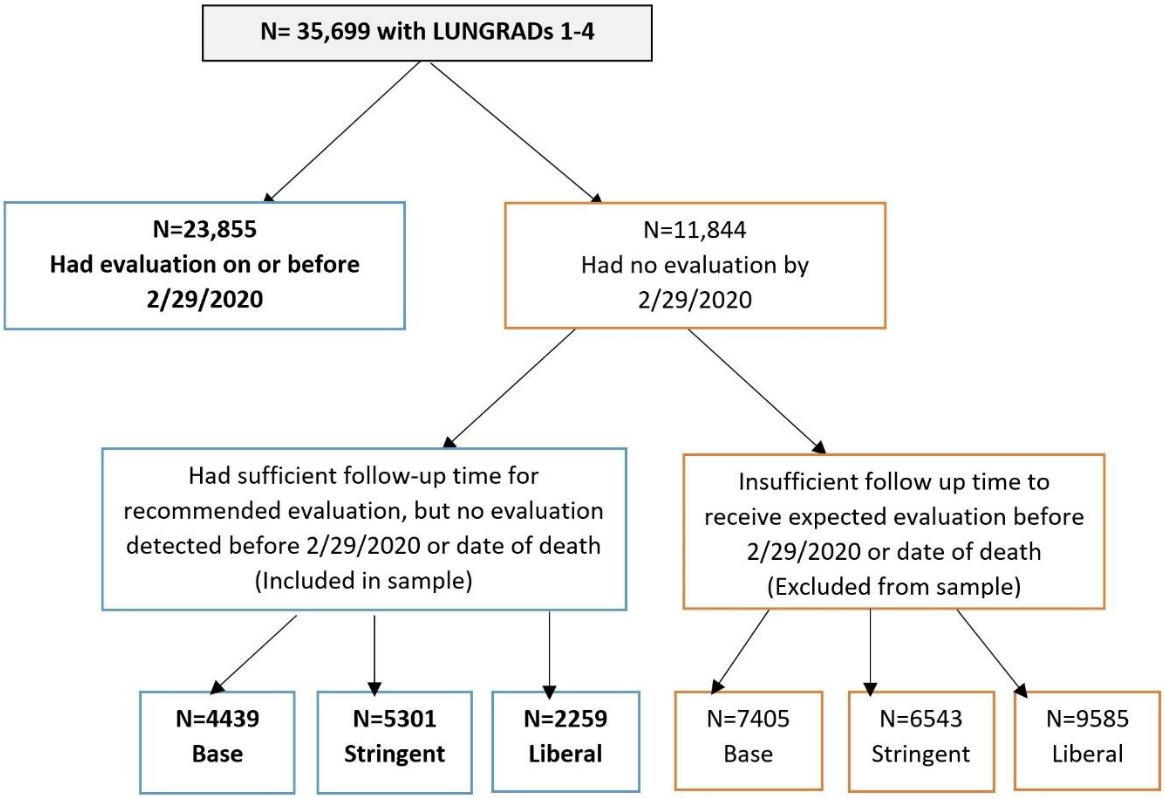


The blue boxes include the analysis samples for each of the three definitions of adherence:*

1. Base model sample: N=23,855+4,439 = 28,294
2. Stringent model sample: N = 23,855+5,301 = 29,156
3. Liberal model sample: N = 23855+2259 = 26,114
4. * Refer to Table 1 for definitions of “early”, “expected” and “late” adherence by Lung-RADS group

© 2021 Núñez ER et al. *JAMA Network Open.*

**eTable 3.** Baseline Characteristics of Veterans Included in Study and Excluded Owing to Insufficient Time to Determine Adherence to Follow-up Testing

| ***Patient characteristics*** | | **N = 28,294** | **N= 7,405** |
|---|---|---|---|
| ***Demographics*** | | | |
| | *Age, yr, mean (SD)* | 65.2 (5.5) | 65.5 (5.8) |
| | *Female sex* | 1,459 (5.2%) | 426 (5.8%) |
| | *Race* | | |
| | White | 21,969 (77.6%) | 5,561 (7.5%) |
| | Black | 5,210 (18.4%) | 1,548 (20.9%) |
| | Hispanic | 602 (2.1%) | 156 (2.1%) |
| | Other | 513 (1.8%) | 140 (1.9%) |
| | *Married* | 12,225 (43.2%) | 3,161 (42.7%) |
| | *Median income based on zip code (IQR)* | $ 46,306 ($18,792) | $44,512 ($17,898) |
| | *Distance from home to LCS facility in miles, median (IQR)* | 28.2 (51.2) | 27.0 (51.8) |
| | *Live in rural zip code* | 6,053 (21.4%) | 1,781 (24.1%) |
| | *VA benefits (priority status)* | | |
| | Highly disabled | 8,143 (28.8%) | 2,313 (31.2%) |
| | Low/moderately disabled | 6,560 (23.2%) | 1,648 (22.3%) |
| | Limited with copayments | 3,698 (13.1%) | 925 (12.5%) |
| | Poverty/no copayments | 9,892 (35.0%) | 2,519 (34.0%) |
| ***Comorbid Conditions*** | | | |
| | Chronic Obstructive Lung Disease | 9,667 (34.2%) | 2,586 (34.9%) |
| | Congestive Heart Failure | 1,731 (6.1%) | 476 (6.4%) |
| | History of major adverse cardiac event | 3,119 (11.0%) | 875 (11.8%) |
| | Chronic kidney disease | 2,918(10.3%) | 759 (10.2%) |
| | Dementia | 731 (2.6%) | 202 (2.7%) |
| | Depression | 7,370 (26.0%) | 2,037 (27.5%) |
| | Anxiety | 3,737 (13.2%) | 1,007 (13.6%) |
| | Post-traumatic stress disorder | 4,701 (16.6%) | 1,258 (17.0%) |
| | Schizophrenia | 706 (2.5%) | 188 (2.5%) |
| | Substance Use disorder | 7,590(26.8%) | 1,960 (26.5%) |
| | Elixhauser Comorbidity Index, mean (SD) | 4.2 (3.1) | 4.2 (3.0) |
| ***Healthcare utilization*** | | | |
| | # Outpatients visit in year before LCS, median (IQR) | 14 (17) | 14 (17) |

© 2021 Núñez ER et al. *JAMA Network Open.*

**eFigure 2.** Individual Facility Odds of Delayed or No Evaluation Stratified by Lung-RADS Group Compared With Lung-RADS 1*

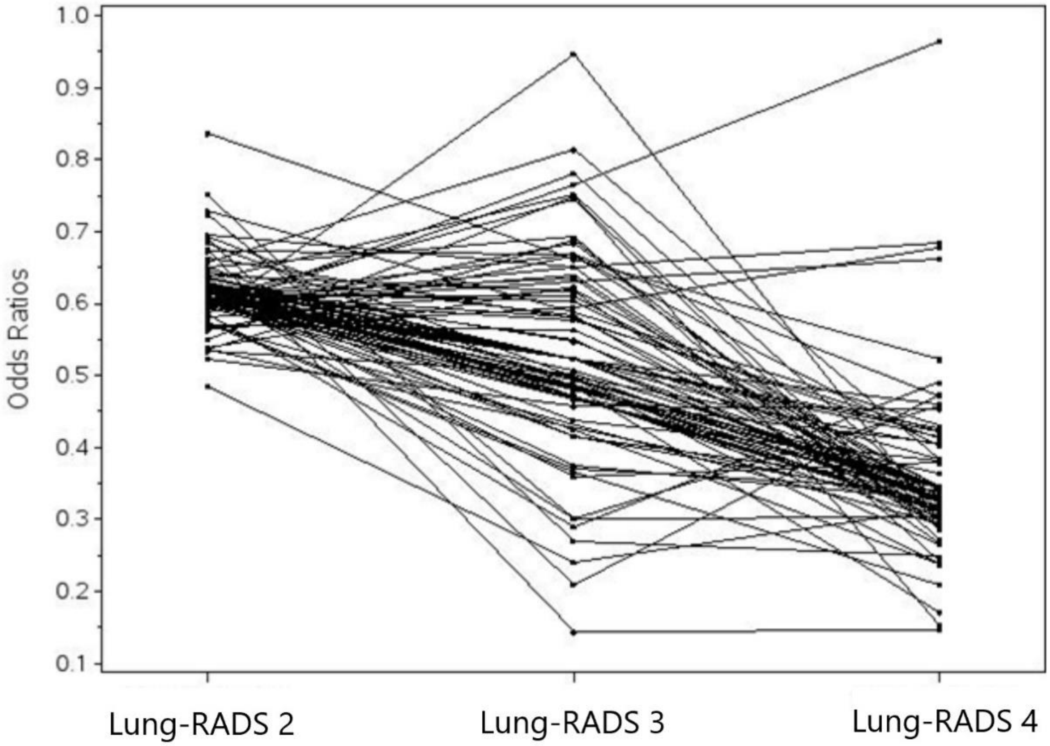


Same facilities connected via solid line.

© 2021 Núñez ER et al. *JAMA Network Open.*

**eTable 4.** Multivariable Logistic Regression Analyses of the Study Cohort Compared With Using Strict and Liberal Adherence Time Cutoffs and Comparing Primary Study Cohort With Low-Risk and High-Risk Separated

| | Base Model (N=27158) | | | Stringent Model (N=27965) | | | Liberal Model (N=25093) | | | Sensitivity Model including only Lung-RADS 1&2(N=20,690) | | | Sensitivity Model including only Lung-RADS 3&4 (N=6,468) | | |
|---|---|---|---|---|---|---|---|---|---|---|---|---|---|---|---|
| **Variables** | **OR** | **95% CI** | | **OR** | **95% CI** | | **OR** | **95% CI** | | **OR** | **95% CI** | | **OR** | **95% CI** | |
| Patient Characteristics | | | | | | | | | | | | | | | |
| **Demographics** | | | | | | | | | | | | | | | |
| Age (years) | 0.98 | 0.97 | 0.98 | 0.98 | 0.97 | 0.98 | 0.98 | 0.97 | 0.99 | 0.98 | 0.97 | 0.98 | 0.98 | 0.97 | 0.99 |
| Female gender | 1.10 | 0.95 | 1.28 | 1.11 | 0.96 | 1.27 | 0.96 | 0.79 | 1.16 | 1.13 | 0.96 | 1.33 | 0.91 | 0.66 | 1.26 |
| Married | 0.89 | 0.83 | 0.94 | 0.90 | 0.86 | 0.95 | 0.88 | 0.81 | 0.96 | 0.91 | 0.85 | 0.97 | 0.80 | 0.68 | 0.95 |
| Rural vs. Urban | 1.01 | 0.93 | 1.10 | 1.02 | 0.95 | 1.10 | 0.92 | 0.82 | 1.03 | 1.02 | 0.93 | 1.12 | 0.96 | 0.80 | 1.15 |
| Distance from home to LCS facility (miles)* | 1.06 | 1.02 | 1.10 | 1.05 | 1.01 | 1.10 | 1.09 | 1.04 | 1.14 | 1.07 | 1.02 | 1.12 | 1.03 | 0.97 | 1.09 |
| Median Income ($)* | 0.88 | 0.79 | 0.98 | 0.94 | 0.85 | 1.03 | 0.88 | 0.77 | 1.01 | 0.90 | 0.80 | 1.01 | 0.82 | 0.66 | 1.03 |
| **Race (white as reference)** | | | | | | | | | | | | | | | |
| Black | 1.19 | 1.10 | 1.29 | 1.13 | 1.04 | 1.21 | 1.24 | 1.12 | 1.38 | 1.20 | 1.10 | 1.32 | 1.14 | 0.95 | 1.37 |
| Hispanic | 1.02 | 0.84 | 1.23 | 0.86 | 0.66 | 1.11 | 0.84 | 0.58 | 1.23 | 1.08 | 0.87 | 1.33 | 0.87 | 0.57 | 1.33 |
| Other | 1.12 | 0.91 | 1.39 | 1.11 | 0.91 | 1.35 | 1.13 | 0.85 | 1.51 | 1.13 | 0.89 | 1.44 | 1.09 | 0.66 | 1.81 |
| **VA benefits (priority status; Veterans with co-pays as reference** | | | | | | | | | | | | | | | |
| Poverty/no-copays | 0.99 | 0.91 | 1.09 | 1.01 | 0.93 | 1.09 | 1.00 | 0.89 | 1.13 | 1.02 | 0.92 | 1.13 | 0.88 | 0.73 | 1.08 |
| Highly disabled | 0.83 | 0.75 | 0.91 | 0.89 | 0.82 | 0.98 | 0.82 | 0.72 | 0.94 | 0.83 | 0.75 | 0.93 | 0.80 | 0.64 | 0.99 |
| Low/moderately disabled | 0.86 | 0.78 | 0.95 | 0.94 | 0.86 | 1.02 | 0.90 | 0.79 | 1.02 | 0.86 | 0.77 | 0.96 | 0.86 | 0.70 | 1.06 |
| **Comorbidities** | | | | | | | | | | | | | | | |
| Elixhauser comorbidity index | 1.01 | 0.99 | 1.02 | 1.00 | 0.99 | 1.01 | 1.01 | 0.99 | 1.03 | 1.00 | 0.99 | 1.02 | 1.04 | 1.00 | 1.07 |
| Major Adverse Cardiac Event | 1.07 | 0.94 | 1.22 | 1.10 | 0.99 | 1.21 | 1.16 | 0.99 | 1.35 | 1.04 | 0.91 | 1.18 | 1.16 | 0.93 | 1.44 |
| Chronic Obstructive Pulmonary Disease | 0.85 | 0.78 | 0.93 | 0.86 | 0.79 | 0.92 | 0.85 | 0.77 | 0.94 | 0.84 | 0.76 | 0.92 | 0.93 | 0.81 | 1.07 |
| Congestive Heart Failure | 0.99 | 0.86 | 1.13 | 1.01 | 0.89 | 1.13 | 0.90 | 0.75 | 1.08 | 1.04 | 0.88 | 1.22 | 0.77 | 0.58 | 1.04 |
| Human Immunodeficiency Virus | 0.88 | 0.59 | 1.31 | 1.16 | 0.87 | 1.54 | 0.84 | 0.51 | 1.39 | 0.78 | 0.51 | 1.19 | 1.71 | 0.71 | 4.12 |
| Chronic kidney disease | 1.02 | 0.90 | 1.16 | 1.05 | 0.94 | 1.18 | 1.05 | 0.91 | 1.21 | 1.04 | 0.90 | 1.21 | 0.96 | 0.76 | 1.22 |
| Dementia | 1.14 | 0.94 | 1.39 | 1.25 | 1.03 | 1.51 | 1.06 | 0.79 | 1.43 | 1.12 | 0.92 | 1.37 | 1.22 | 0.84 | 1.77 |
| Schizophrenia | 1.12 | 0.92 | 1.36 | 1.12 | 0.95 | 1.32 | 1.23 | 0.96 | 1.59 | 1.11 | 0.89 | 1.39 | 1.09 | 0.72 | 1.63 |
| Post-traumatic stress disorder | 1.13 | 1.03 | 1.23 | 1.06 | 0.98 | 1.15 | 1.19 | 1.06 | 1.34 | 1.11 | 1.00 | 1.22 | 1.23 | 1.00 | 1.50 |
| Depression | 1.06 | 0.98 | 1.15 | 1.11 | 1.03 | 1.20 | 1.08 | 0.98 | 1.20 | 1.08 | 0.98 | 1.18 | 1.01 | 0.85 | 1.20 |

© 2021 Núñez ER et al. *JAMA Network Open.*

| | | | | | | | | | | | | | | | |
|---|---|---|---|---|---|---|---|---|---|---|---|---|---|---|---|
| Anxiety | 1.04 | 0.95 | 1.14 | 1.05 | 0.96 | 1.14 | 1.00 | 0.89 | 1.13 | 1.03 | 0.93 | 1.14 | 1.08 | 0.88 | 1.32 |
| Substance use disorder | 1.11 | 1.01 | 1.22 | 1.13 | 1.04 | 1.22 | 1.16 | 1.03 | 1.30 | 1.12 | 1.01 | 1.24 | 1.11 | 0.94 | 1.30 |
| **Healthcare utilization** | | | | | | | | | | | | | | | |
| # Outpatient visits in year before LCS* | 0.86 | 0.82 | 0.91 | 0.86 | 0.82 | 0.90 | 0.83 | 0.77 | 0.89 | 0.88 | 0.83 | 0.93 | 0.79 | 0.71 | 0.89 |
| **Lung-RADS category (LungRADS-1 as reference)** | | | | | | | | | | | | | | | |
| LungRADS-2 | 0.62 | 0.56 | 0.68 | 0.65 | 0.59 | 0.71 | 0.51 | 0.44 | 0.59 | 0.61 | 0.56 | 0.68 | 0.65 | 0.52 | 0.80 |
| LungRADS-3 | 0.52 | 0.43 | 0.64 | 0.49 | 0.42 | 0.58 | 1.02 | 0.82 | 1.26 | | | | | | |
| LungRADS-4 | 0.35 | 0.28 | 0.43 | 0.28 | 0.23 | 0.33 | 0.78 | 0.62 | 0.98 | | | | | | |
| Facility Characteristics | | | | | | | | | | | | | | | |
| Academic | 0.86 | 0.80 | 0.93 | 0.96 | 0.89 | 1.02 | 0.90 | 0.81 | 0.99 | 0.83 | 0.76 | 0.90 | 1.00 | 0.85 | 1.17 |
| Thoracic surgery | 1.08 | 0.64 | 1.83 | 0.92 | 0.54 | 1.58 | 0.87 | 0.47 | 1.63 | 1.11 | 0.62 | 2.00 | 0.77 | 0.44 | 1.35 |
| **Geographic location (Northeast as reference** | | | | | | | | | | | | | | | |
| Midwest | 1.16 | 0.58 | 2.33 | 1.31 | 0.64 | 2.70 | 0.82 | 0.36 | 1.86 | 1.42 | 0.65 | 3.11 | 1.10 | 0.53 | 2.31 |
| South | 1.01 | 0.54 | 1.89 | 1.22 | 0.64 | 2.33 | 0.53 | 0.25 | 1.12 | 1.09 | 0.54 | 2.20 | 1.24 | 0.65 | 2.35 |
| West | 0.77 | 0.38 | 1.55 | 0.94 | 0.46 | 1.91 | 0.57 | 0.25 | 1.31 | 0.78 | 0.35 | 1.70 | 1.15 | 0.56 | 2.38 |
| **# of LCS performed (reference < 500)** | | | | | | | | | | | | | | | |
| 500-1000 | 0.65 | 0.41 | 1.04 | 0.74 | 0.45 | 1.22 | 0.85 | 0.48 | 1.53 | 0.67 | 0.40 | 1.12 | 0.66 | 0.42 | 1.04 |
| > 1000 | 0.38 | 0.21 | 0.67 | 0.51 | 0.29 | 0.90 | 0.52 | 0.25 | 1.08 | 0.36 | 0.19 | 0.70 | 0.63 | 0.36 | 1.10 |

© 2021 Núñez ER et al. *JAMA Network Open.*
